# A non-smooth tumor margin on preoperative imaging assesses microvascular invasion of hepatocellular carcinoma: A systematic review and meta-analysis

**DOI:** 10.1038/s41598-017-15491-6

**Published:** 2017-11-13

**Authors:** HangTong Hu, Qiao Zheng, Yang Huang, Xiao Wen Huang, Zhi Cheng Lai, JingYa Liu, XiaoYan Xie, Shi Ting Feng, Wei Wang, Ming De Lu

**Affiliations:** 1 Department of Medical Ultrasonics, Institute of Diagnostic and Interventional Ultrasound, The First Affiliated Hospital of Sun Yat-Sen University, Guangzhou, China; 2 Department of Radiology, the First Affiliated Hospital of Sun Yat-Sen University, Guangzhou, China; 3 Department of Hepatobiliary Surgery, The First Affiliated Hospital of Sun Yat-Sen University, Guangzhou, China

## Abstract

Microvascular invasion (MVI) is rarely diagnosed preoperatively in hepatocellular carcinoma (HCC). The aim of this meta-analysis is to assess the diagnostic power of a non-smooth tumor margin on preoperative imaging for MVI. We performed a literature search using the PubMed, Embase and Cochrane Library databases, and 11 studies were included involving 618 MVI-positive cases and 1030 MVI-negative cases. Considerable heterogeneity was found, and was indicated to be attributable to the mean patient ages in the included studies. In subgroups of studies with a mean patient age older than 60 years and studies with computed tomography (CT) as the imaging method (as opposed to magnetic resonance imaging (MRI)), heterogeneity was low, and the diagnostic odds ratio (DOR) of the single two-dimensional imaging feature for MVI was 21.30 (95% CI [12.52, 36.23]) and 28.78 (95% CI [13.92, 59.36]), respectively; this power was equivalent to or greater than that of certain multivariable-based scoring systems. In conclusion, a non-smooth tumor margin on preoperative imaging is of great value for MVI assessment and should be considered for inclusion in future scoring systems.

## Introduction

Hepatocellular carcinoma (HCC) is the third-leading cause of cancer-related deaths worldwide^1^; even with treatment with potentially curative therapies, HCC has a 5-year recurrence rate of approximately 35–70%^2^. Vascular invasion is direct evidence of the biological aggressiveness of a tumor. Microvascular invasion (MVI), which is defined as tumor invasion of a portal vein radicle, the large capsule vessel or a vascular space lined by endothelial cells, is detectable only by microscopy^3^. Relative to other HCC patients, patients with MVI have a markedly higher risk of early recurrence and poor postsurgical survival^3,4^, even in cases involving small, solitary HCC tumors^5,6^. Moreover, patients who do not satisfy the Milan criteria can nonetheless exhibit excellent outcomes if they are confirmed to be negative for MVI^7^.

The gold standard for diagnosing MVI is a histological examination, which requires extensive sampling. A noninvasive evaluation system capable of preoperatively identifying MVI would be of great clinical value for better determining optimal therapeutic strategies. If MVI is detected, an adjuvant therapy such as sorafenib treatment^8^ or trans-arterial chemoembolization (TACE)^9^ could be used, given that these approaches have been reported to improve survival for HCC patients with MVI.

Several promising markers of MVI have been identified, although assessments of such markers involve technically demanding methods, including genetic testing, protein analysis and radiographic examination. A non-smooth tumor margin, which manifests as a lobulated or irregularly shaped tumor with the focal/multifocal outgrowth of nodules protruding into the non-tumor parenchyma^10,11^, is a two-dimensional imaging feature observable via even the simplest imaging methods, including ultrasonography. Moreover, this feature has been reported to be strongly related to elevated MVI risk and has even been identified as the only significant factor in a multivariable analysis^10,12–14^. To better understand this topic, we performed a review of the diagnostic performance of a non-smooth tumor margin for preoperative MVI assessment.

## Results

### Study selection

**Figure 1:**
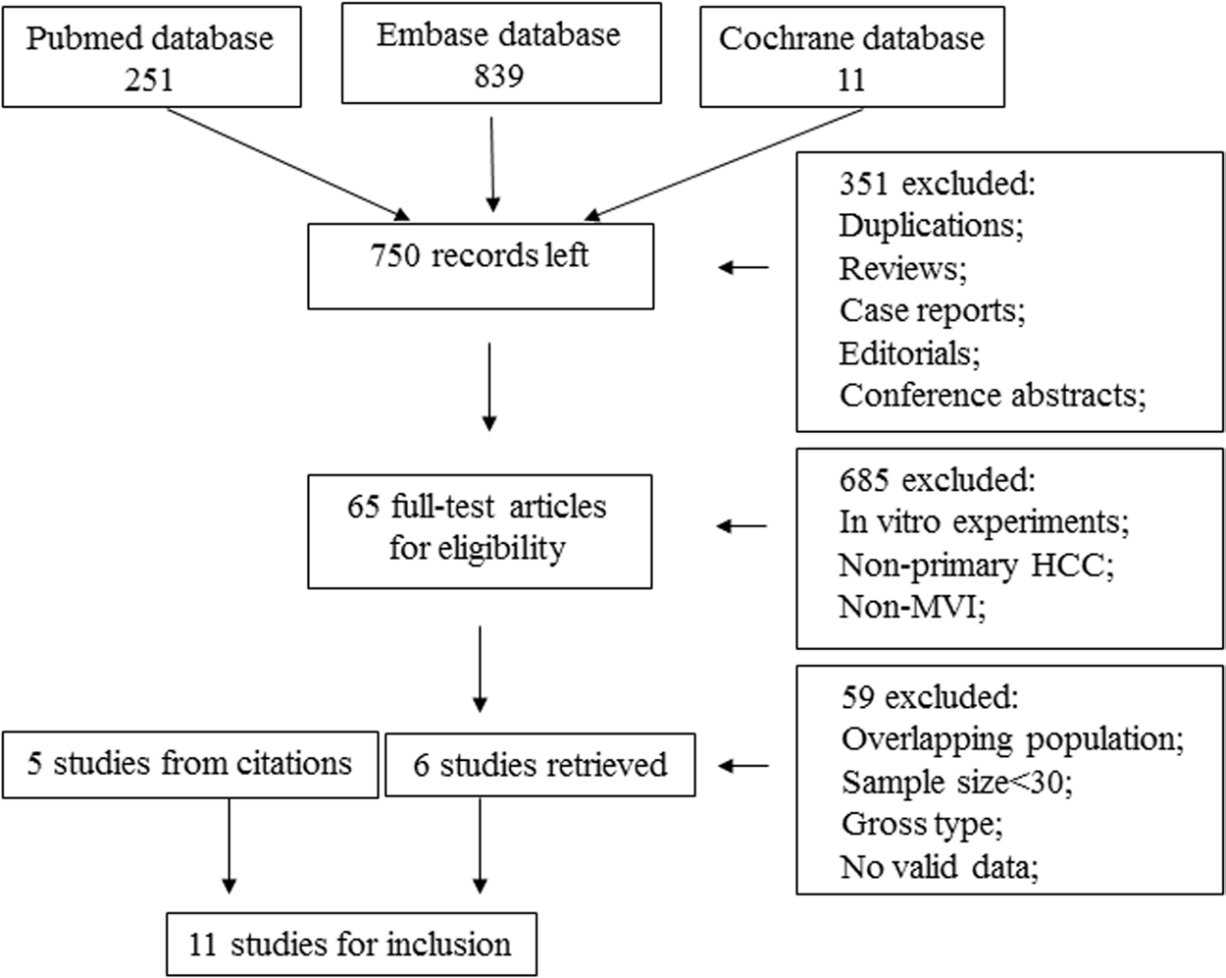
Flow chart diagram presenting the selection of eligible studies.

We identified a total of 1101 studies using our search strategy (Fig. 1). Studies as duplicated reports, reviews, case reports, editorials or conference abstracts were excluded (351 studies). After titles and abstracts were reviewed, 685 studies were excluded for examining non-primary HCC, being *in vitro* experiments or unrelated to MVI estimation. The remaining 65 studies were subjected to full-text review, and 59 of these studies were excluded for overlapping population (3 studies), sample size smaller than 30 (1 study), involving gross analyses of non-smooth tumor margins (7 studies), or containing no valid extractable data for our meta-analysis (48 studies). Six studies were selected for inclusion, and 5 additional studies were included after reviewing citations of the retrieved articles.

### Study characteristics

Characteristics of the 11 studies are presented in Table 1, and additional information is presented in Table 1 of the Supplementary materials. The included studies were published between 2009 and 2016. In total, 618 pathologically diagnosed MVI-positive patients and 1030 MVI-negative patients were included in this meta-analysis. Two^15,16^ of the included studies were performed in European populations, and the remaining nine studies were performed in Asian populations. One study^17^ assessed HCC patients treated via liver transplantation, 8 studies assessed HCC patients treated via liver resection, and two^10,16^ studies included HCC patients treated via either modality. Tumor margins were evaluated using computed tomography (CT), magnetic resonance imaging (MRI), and either of these approaches in two studies^10,12^, 7 studies, and two studies^14,15^, respectively. In Witjes *et al*.^16^, 22% of patients received preoperative radiofrequency ablation (RFA) or trans-arterial chemoembolization (TACE). Patients with macrovascular invasion and patients who received preoperative anti-tumor treatment were excluded from the remaining studies, with the exception of the study by Kim *et al*.^11^, which did not clearly address this issue.

**Table 1:** Characteristics of the 11 included studies.

Study	Year	Country	Patients(Tumors) (n)	Age	Imaging methods	P (non-smooth) (%)	TP	FP	FN	TN	QUADAS Score
Kim *et al*.^11^	2009	Korea	66(70)	55	MR	39	19	8	16	27	11
Ariizumi *et al*.^13^	2011	Japan	61(61)	67	MR	39	10	14	1	36	9
Chou *et al*.^12^	2012	China	102(102)	60	CT	39	33	7	17	45	13
Witjes *et al*.^16^	2012	Netherlands	64(64)	56	MR	28	14	4	31	15	6
Chou *et al*.^10^	2014	China	102(102)	63	CT	53	49	5	11	37	12
Xu *et al*.^29^	2014	China	109(109)	53	MR	18	8	12	31	58	13
Ahn *et al*.^17^	2015	Korea	51(78)	52	MR	40	11	20	7	40	11
Lei *et al*.^19^	2016	China	707(707)	52	MR	17	33	90	178	406	10
Renzulli *et al*.^15^	2016	Italy	125(140)	62	CT/MR	59	75	8	15	42	8
Wu *et al*.^14^	2016	Japan	79(79)	70	CT/MR	38	13	17	2	47	9
Yang *et al*.^18^	2016	China	136(136)	56	MR	39	24	29	20	63	7

Patients (Tumors) (n): TP, FP, FN and TN referred to tumor number. Number outside the bracket was that of patients, and inside the bracket was that of tumor number; MR: Magnetic Resonance Imaging; CT: Computed Tomography; P (non-smooth) (%): percentage of patients with a non-smooth tumor margin on preoperative imaging test; TP: true-positive, FP: false-positive, FN: false-negative, TN: true-negative.

### Heterogeneity test and meta-regression analysis

**Figure 2:**
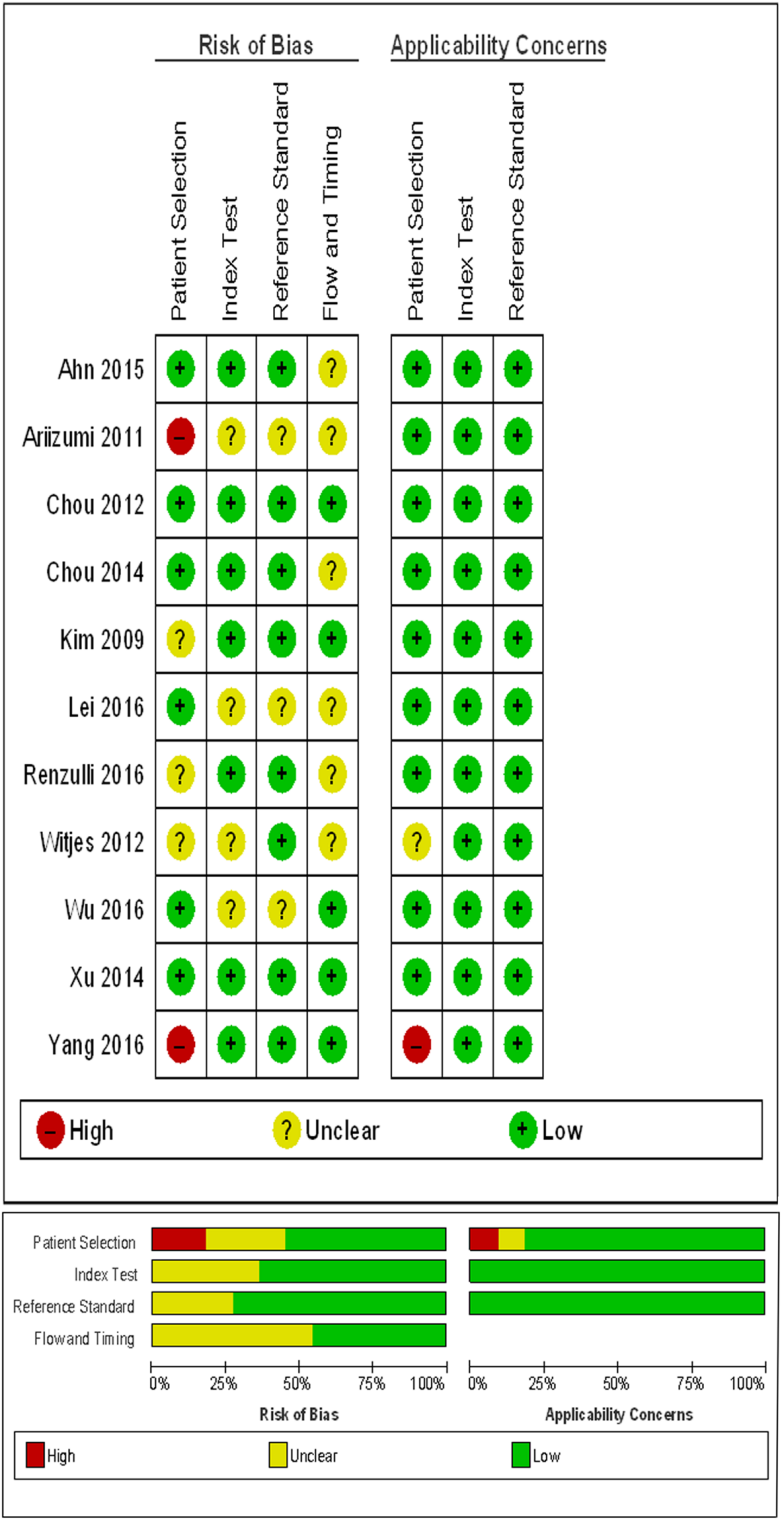
Quality of included studies according to QUADAS-2 guidelines. Risk of bias and applicability concerns of each included study. Proportion of studies with risk of bias; proportion of studies with regarding applicability. The risk bias mainly raised from patient selection due to inappropriate inclusion and exclusion, and determined as “high”. Another source of bias was indefinite report of blind method and interval between imaging and MVI pathological detection, and determined as “unclear”.

The Spearman correlation coefficient was 0.06, and the corresponding *P* value was 0.85, suggesting the absence of threshold effect-derived heterogeneity. However, the inconsistency indexI^2^ was 89.1%, indicating heterogeneity due to non-threshold effects. Meta-regression analysis indicated that the patients’ mean age may have contributed to this high heterogeneity. QUADAS score summaries are presented in Table 1 and Fig. 2. The QUADAS score varies from 6 to 13, which indicated that other potential contributors to the heterogeneity resulted from the risk bias in patient selection due to the inappropriate inclusion and exclusion of patients (Ariizumi *et al*.^13^ included patients of recurrent HCC with a history of hepatectomy; and Yang *et al*.^18^ excluded patients with interval between MRI and surgery longer than 14 days). This risk bias may indirectly impact on the mean age and patient percentage with a positive imaging parameter. We adopted a clinical perspective and conducted sensitivity and subgroup analyses based on the patients’ mean age, with a threshold of 60 years; imaging method; and the percentage of patients with a non-smooth tumor margin.

### Accuracy of a non-smooth tumor margin for predicting MVI

**Figure 3:**
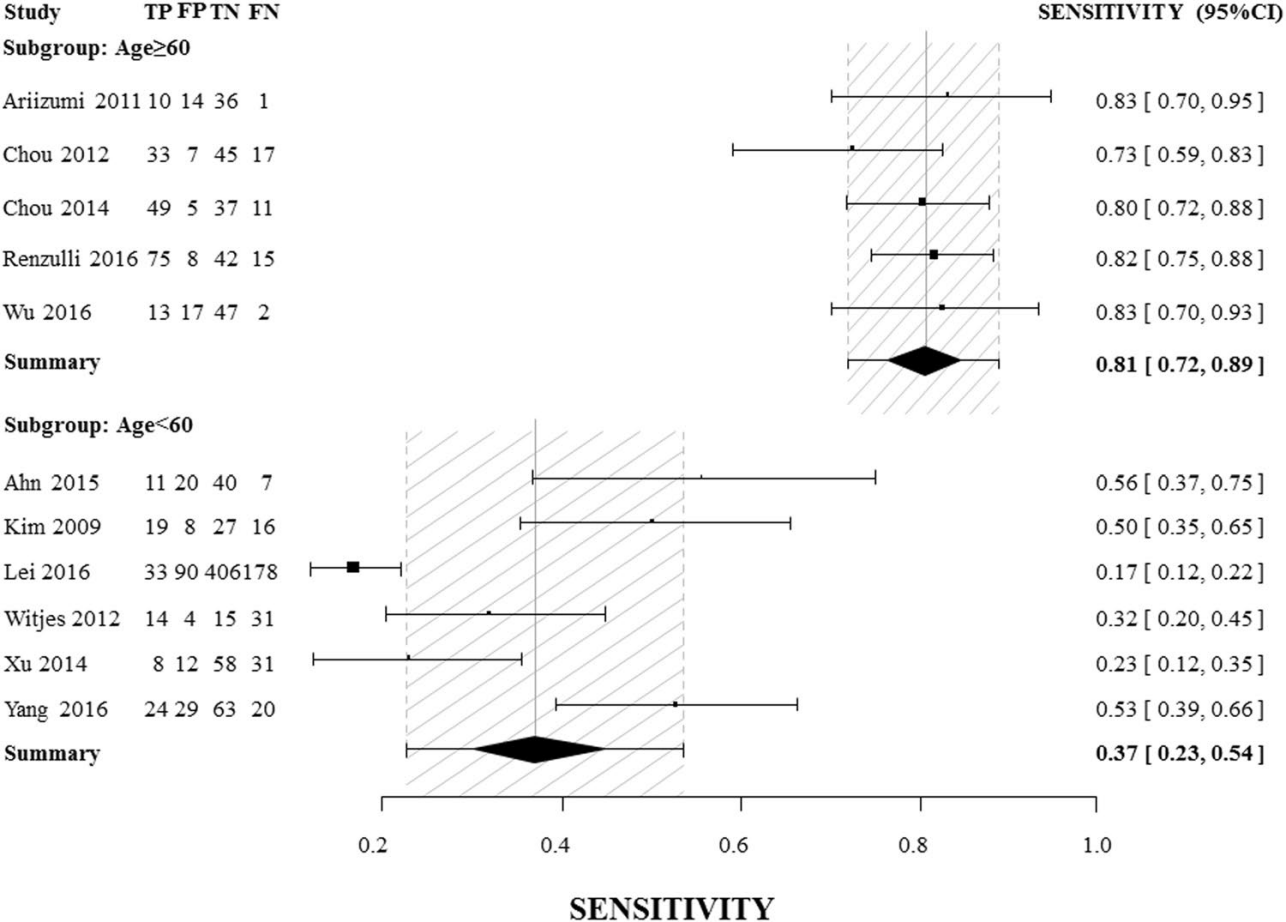
Forest plots of sensitivity and exact 95% confidence interval for subgroups of age ≥ 60 and age <60. CI: confidence interval. TP: true-positive, FP: false-positive, FN: false-negative, TN: true-negative. The summary sensitivity was 0.81[0.72, 0.89] for the subgroup of age older than 60, and 0.37[0.23, 0.54] for that of age younger than 60.

**Figure 4:**
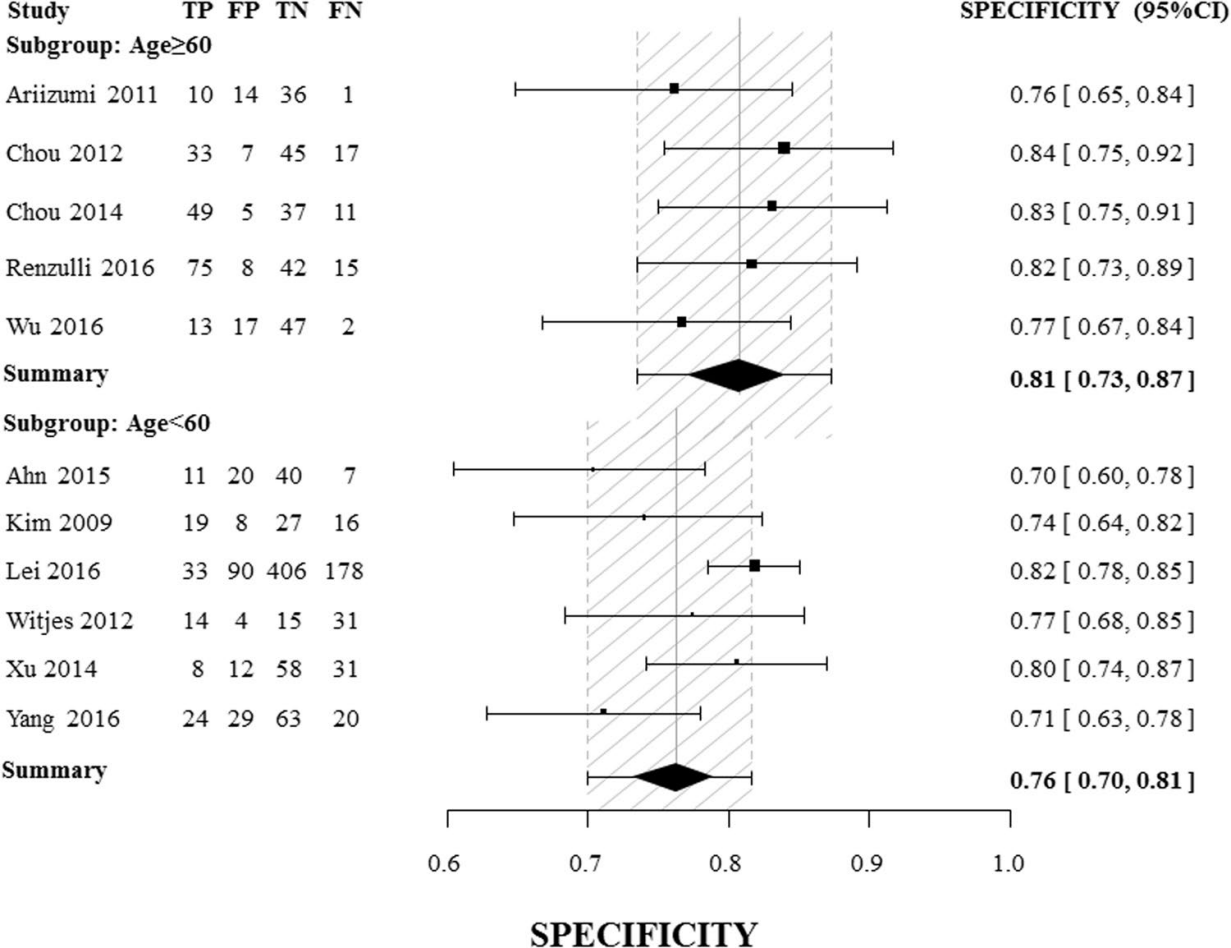
Forest plots of specificity and exact 95% confidence interval for subgroups of age ≥ 60 and age <60. CI: confidence interval. TP (true-positive), FP (false-positive), FN (false-negative), TN (true-negative). The summary specificity was 0.81[0.73, 0.87] for the subgroup of age older than 60, and 0.76[0.70, 0.81] for that of age younger than 60.

High heterogeneity among studies precluded the pooling of data to obtain a summary result. As indicated in Table 2, subgroup analysis stratified by age with a threshold of 60 years showed that a non-smooth tumor margin has strong diagnostic power for MVI in studies with a mean patient age older than 60. In particular, the DOR of a non-smooth tumor margin could reach 21.30 (95% CI [12.52, 36.23]), with an area under the curve (AUC) as high as 0.90. And the summary sensitivity and specificity of the subgroup of age older than 60 was 0.81[0.72, 0.89] and 0.81[0.73, 0.87], respectively (Fig. 3 and Fig. 4). A non-smooth tumor margin had stronger diagnostic power when CT was used for imaging (2 studies) than when MRI was the imaging approach, and strong diagnostic power was also observed in the studies which included greater than 53% patients with a non-smooth tumor margin (2 studies), with DORs in this case reaching 19.42 (95% CI [7.53, 50.16]) and 28.78 (95% CI [13.92, 59.36]), respectively. Table 3 presents a comparison between using the single factor of a non-smooth tumor margin and using multivariable scoring systems for MVI assessment. Six scoring systems were identified^19–24^, and the comparison indicated that for the subgroups of patients older than 60 and patients who underwent CT imaging, a non-smooth tumor margin exhibited excellent diagnostic power. This power was equivalent to or greater than the diagnostic powers calculated for certain multivariable scoring systems.

**Table 2:** Pooled results of subgroup analysis.

Category		SEN[95%CI]	SPE[95%CI]	PLR[95%CI]	NLR[95%CI]	DOR[95%CI]	AUC
Age (mean)	≥60	0.81[0.72, 0.89]	0.81[0.73, 0.87]	4.14[2.99, 5.74]	0.25[0.17, 0.37]	21.30[12.52, 36.23]	0.90
	<60	0.37[0.23, 0.54]	0.76[0.70, 0.81]	1.47[1.03, 2.09]	0.82[0.67, 1.01]	1.84[1.02, 3.34]	0.72
Imaging	CT	0.75[0.65, 0.82]	0.87[0.79, 0.93]	5.66[3.29, 9.74]	0.29[0.16, 0.55]	19.42[7.53, 50.16]	—
	MR	0.30[0.25, 0.34]	0.78[0.75, 0.81]	1.68[1.12, 2.53]	0.79[0.62, 0.99]	2.25[1.15, 4.38]	0.74
P (non-smooth) (%)	≥53	0.83[0.76, 0.88]	0.86[0.77, 0.92]	5.77[3.47, 9.59]	0.20[0.14, 0.29]	28.78[13.92, 59.36]	—
	<53	0.35[0.31, 0.40]	0.79[0.76, 0.81]	2.03[1.36, 3.03]	0.65[0.48, 0.88]	3.51[1.62, 7.58]	0.79

SEN: sensitivity; SPE: specificity; PLR: positive likelihood ratio; NLR: negative likelihood ratio; DOR: diagnostic odds ratio; AUC: area under the receiver operating characteristic curve.

**Table 3:** Comparisons with multivariable based scoring system.

Items	Factors	Category	SEN	SPE	PLR	NLR	DOR	AUC
Non-smooth tumor margin	Single factor	Age < 60	0.81	0.81	4.14	0.25	21.30	0.90
		CT	0.75	0.87	5.66	0.29	19.42	—
Lei *et al*.^19^	Tumor size + Tumor number + AFP + HBV DNA load + PLT + Typical dynamic pattern + Tumor capsule	Age = 52	0.62	0.81	3.20	0.47	7.0^	0.80
Liu *et al*.^20^	AFP + miR125b	Age = 54	0.84	0.72	3.00^	0.22^	13.5^	0.87
Miyata *et al*.^21^	T1 ring + AP shunt + Distortion of corona	Age = 67	0.82	0.84	5.13^	0.21^	23.9^	—
Shirabe *et al*.^22^	Tumor size + DCP + Tumor grade	Age = 61	0.75	0.85	5.00^	0.29^	17.0^	—
You *et al*.^23^	Tumor size + AFP + Hypersplenism	Age = 50	0.76	0.75	3.04^	0.32^	9.50^	0.79
Zhao *et al*.^30^	AFP + GGT + Tumor size + Tumor number	Age = 50	0.82	0.83	4.8	0.2	22.24^	0.86

^Data was obtained by estimation with SEN and SPE by DOR = SEN*SPE/(1-SEN)/(1-SPE), or PLR = SEN/(1-SPE), or NLR = (1-SEN)/SPE.

## Discussion

Preoperative MVI assessment has become clinically possible given the discovery of associations between a pathological diagnosis of MVI and imaging/clinical features, proteomics characteristics and gene signatures^2,25–27^. Pathologically, MVI has been reported to most frequently present at the site of extranodal extension^10,12^. Moreover, a non-smooth tumor margin can be regarded as indicative of HCC’s aggressive biological tendencies to invade the tumor capsule and protrude into the non-tumoral parenchyma. In this meta-analysis, we systematically reviewed studies evaluating the diagnostic accuracy of a non-smooth tumor margin for MVI. High heterogeneity among the included studies precluded the pooling of results to draw clinically applicable findings. However, the subgroups of studies with a mean patient age older than 60 years and studies involving CT imaging exhibited low heterogeneity and indicated that the diagnostic power of a non-smooth tumor margin for MVI was strong. This diagnostic power was found to be equivalent to or even greater than the diagnostic powers of certain multivariable-based scoring systems. This result shows that a non-smooth tumor margin is a promising indicator of preoperative MVI assessment. The diagnostic accuracy for MVI may be further improved by constructing a model that includes a non-smooth tumor margin in addition to other factors.

Several considerations warrant attention. First, image interpretation is equipment dependent. Our study reveals that CT is superior to MRI with respect to evaluating a non-smooth tumor margin for MVI assessment. This result is reasonable given that CT has better spatial resolution than MRI and that spatial resolution is extremely important for distinguishing between non-smooth and smooth tumor margins. However, only 2 studies from the same research group (with different patient populations) addressed the use of CT to evaluate tumor margins for MVI assessment; therefore, the pooled result is incompletely convincing, and more high-quality evidence is needed to reach more definitive conclusions. One possible explanation for why the subgroup of studies with a mean patient age older than 60 years exhibited relatively good pooled results is that CT was used for imaging at a higher rate in these studies than in studies involving younger patients (82% and 0%, respectively). Our study revealed that CT was a better assessment approach than MRI in the context of tumor margin evaluation, although ultrasonography may also play an important role given its advantage of enabling the scanning of additional sections compared to CT and MR. In our opinion, the intercostal or subcostal scanning of ultrasonography can obtain sections along multiple axis of the tumor, which is superior in examining irregular shaped tumors. Moreover, ultrasonography is economical and does not result in radiation exposure. However, to date, no published study has used ultrasonography for MVI-related assessments of tumor margins.

The results related to patients with a non-smooth tumor margin larger than 53% point to the most important limitation of our analysis. Image interpretation is both equipment dependent and operator dependent. Yamashita *et al*.^6^ reported that relative to pathological diagnosis, imaging diagnosis of a non-smooth tumor margin has a sensitivity of only 54%. Controversy exists with respect to evaluations of a non-smooth tumor margin. In 2 studies with nearly the same eligibility criteria, Chou *et al*.^10,12^ reported finding a non-smooth tumor margin in different percentages (53% and 39%, respectively) of patients from the same ethnic group, indicating that tumor margins may be misjudged and that the association of a non-smooth tumor margin with MVI risk may have been underestimated (odds ratios of 33.0 and 12.5, respectively). This possibility is consistent with the results of our meta-analysis. There are several additional limitations of the present study. First, considerable heterogeneity among studies was observed. Clinical heterogeneity (for example, ethnographic characteristics) may limit the reliability of pooled results. Moreover, a limited number of studies were included in this meta-analysis, and most of these studies had small sample sizes. Additional large-scale and high-quality studies are necessary to reach more definitive conclusions. In summary, our study reveals that a non-smooth tumor margin is a promising two-dimensional imaging feature for the preoperative assessment of MVI. Further investigation of this association is required, and this feature should be considered in the construction of future scoring systems.

## Methods

### Search strategy

Pertinent studies were identified by searching the PubMed, Embase, and Cochrane Library databases for articles published by April 12, 2017. No language or other restrictions were imposed. This search involved the use of “hepatocellular carcinoma” as a MeSH term and a free term and “microvascular invasion” as a free term combined with the search terms “margin”, “boundary”, “irregular”, “lobulated”, “confluence”, “infiltrative” and “extension”. The full search strategy is described in the Supplementary materials.

### Inclusion and exclusion criteria

Clinical trials addressing the use of a non-smooth tumor margin for the preoperative evaluation of MVI in HCC patients were considered for inclusion. Studies were required to report sufficient data to construct a diagnostic 2 × 2 table. Investigations of non-primary HCC, studies involving gross analyses (without imaging) of non-smooth tumor margins and trials with sample sizes smaller than 30 subjects were excluded. When multiple publications assessed the same population, only data from the most recent comprehensive report were included. A literature flow diagram is depicted in Fig. 1.

### Data extraction and quality assessment

All identified studies were screened for eligibility by one author (Y.H.), and a sample of 20% of these studies was independently assessed by another author (X.W.H.) to ensure consistent application of the eligibility criteria. Potentially eligible citations from retrieved articles were reviewed to identify additional studies. Both investigators (Y.H. and X.W.H.) used QUADAS (Quality Assessment of Diagnostic Accuracy Studies included in systematic reviews) to evaluate level of evidence and reached consensus^28^. Two authors (H.T.H. and Q.Z.) independently extracted the following data from each included study: the first author’s last name, publication year, country, sample size, mean age and possible sources of bias. These variables included mean tumor size, the percentage of male or MVI-positive patients and patients with a non-smooth tumor margin, the inclusion of patients with macrovascular invasion, the administration of preoperative anti-tumor treatment, surgical modality (i.e., resection or transplantation) and imaging modality. Data were extracted as true positives (TP), false positives (FP), false negatives (FN) and true negatives (TN) to form a diagnostic 2 × 2 table. Disagreements during study selection, data extraction and quality assessment were resolved by consensus, with arbitration by a third author as needed (W.W.).

### Statistical analysis

We used R (version 3.2.5; The R Foundation for Statistical Computing, Vienna, Austria) and Meta-Disc 1.4 for Windows (XI Cochrane Colloquium, Barcelona, Spain) for all statistical analyses. Summary results are presented in terms of sensitivity (SEN), specificity (SPE), positive likelihood ratio (PLR), negative likelihood ratio (NLR), diagnostic odds ratio (DOR) and area under the receiver operating characteristic curve (AUC-ROC). Heterogeneity due to the threshold effect was investigated using the Spearman correlation coefficient in Meta-Disc 1.4. A strong positive correlation (*P* < 0.05) suggested a significant threshold effect. Heterogeneity caused by non-threshold effects was measured using the inconsistency index I^2^, with I^2^ < 50% indicating heterogeneity among studies. In cases involving heterogeneity, the DerSimonian-Laird method was used to calculate estimates. A meta-regression was performed to evaluate possible sources of bias listed in the Supplementary material (Table 1) and QUADAS scores, which were the basis for subgroup analyses. All tests of statistical significance were two-sided, and the chosen threshold for statistical significance was 0.05.

## Electronic supplementary material

Supplementary materials
