# Supplementary material for: A non-smooth tumor margin on preoperative imaging assesses microvascular invasion of hepatocellular carcinoma: A systematic review and meta-analysis
Source: Sci Rep. 2017 Nov 13;7:15375. doi: 10.1038/s41598-017-15491-6 (PMC5684346; doi:10.1038/s41598-017-15491-6)
Supplement: Supplementary file 1 — Supplementary materials [file 41598_2017_15491_MOESM1_ESM.docx]

**A non-smooth tumor margin on preoperative imaging assesses microvascular invasion of hepatocellular carcinoma: A systematic review and meta-analysis**

HangTong Hu^1#^, Qiao Zheng^1#^, Yang Huang^1^, XiaoWen Huang^1^, ZhiCheng Lai^1^, JingYa Liu^1^, XiaoYan Xie^1^, ShiTing Feng^2*^, Wei Wang^1*^, MingDe Lu^1,3^.

^1^Department of Medical Ultrasonics, Institute of Diagnostic and Interventional Ultrasound, The First Affiliated Hospital of Sun Yat-Sen University, Guangzhou, China.

^2^Department of Radiology, the First Affiliated Hospital of Sun Yat-Sen University, Guangzhou, China.

^3^Department of Hepatobiliary Surgery, The First Affiliated Hospital of Sun Yat-Sen University, Guangzhou, China.

NO.58 Zhongshan Road 2, Guangzhou, 510080, People’s Republic of China.

Contents

[Search strategy 3](#_Toc481105258)

[Pubmed 3](#_Toc481105259)

[Embase 4](#_Toc481105260)

[Cochrane Library 5](#_Toc481105261)

[Table 1: Detailed characteristics of the 11 included studies 6](#_Toc481105262)

[Table 2: Results of Meta-regression 7](#_Toc481105263)

[Table 3: Heterogeneity (I^2^) of pooled results 7](#_Toc481105264)

[References: 8](#_Toc481105265)

#

# ****Search strategy****

## **Pubmed**

1. **hepatocellular carcinoma: ("Carcinoma, Hepatocellular"[Mesh]) OR ((((((((((((((((((Carcinomas, Hepatocellular[Title/Abstract]) OR Hepatocellular Carcinomas[Title/Abstract]) OR Liver Cell Carcinoma, Adult[Title/Abstract]) OR Liver Cancer, Adult[Title/Abstract]) OR Adult Liver Cancer[Title/Abstract]) OR Adult Liver Cancers[Title/Abstract]) OR Cancer, Adult Liver[Title/Abstract]) OR Cancers, Adult Liver[Title/Abstract]) OR Liver Cancers, Adult[Title/Abstract]) OR Liver Cell Carcinoma[Title/Abstract]) OR Carcinoma, Liver Cell[Title/Abstract]) OR Carcinomas, Liver Cell[Title/Abstract]) OR Cell Carcinoma, Liver[Title/Abstract]) OR Cell Carcinomas, Liver[Title/Abstract]) OR Liver Cell Carcinomas[Title/Abstract]) OR Hepatocellular Carcinoma[Title/Abstract]) OR** **Hepatoma[Title/Abstract]) OR Hepatomas[Title/Abstract]);**
2. **microvascular invasion:** **(((portal vein[Title/Abstract] OR microvascular[Title/Abstract] OR microvessel[Title/Abstract] OR microscopic[Title/Abstract])) AND (invasion[Title/Abstract] OR emboli[Title/Abstract] OR thrombi[Title/Abstract] OR thrombosis[Title/Abstract])) OR (microemboli[Title/Abstract] OR microthrombi[Title/Abstract] OR microthrombosis[Title/Abstract]);**
3. **non-smooth tumor margin:** **((((((margin) OR boundary) OR irregular) OR lobula*) OR confluen*) OR infiltrat*) OR extension;**
4. **Final search strategy: ① AND ② AND ③;**
5. **Final record: 251 studies.**

## **Embase**

**#1** **'liver cell carcinoma'/exp OR 'liver cell carcinoma'**

**#2** **liver or hepatic or hepatocellular:ab,ti**

**#3** **neoplasm or cancer or carcinoma or tumor:ab,ti**

**#4** **HCC or hepatoma:ab,ti**

**#5** **#2 AND #3 OR # 1 OR #4**

**#6** **microvascular or microvessel or microscopic or "portal vein":ab,ti**

**#7** **invasion or emboli or thrombi or thrombosis:ab,ti**

**#8** **microinvasion or microemboli or microthrombi or microthrombosis:ab,ti**

**#9 #6 And #7 OR #8**

**#10** **margin OR boundary OR irregular OR lobula* OR confluen* OR extension OR infiltrat***

**#11 #5 AND #9 AND #10**

**Final record: 839 studies.**

## **Cochrane Library**

**#1: MeSH descriptor: [****Carcinoma, Hepatocellular]**

**#2: liver or hepatic or hepatocellular:ti,ab,kw**

**#3: neoplasm or cancer or carcinoma or tumor:ti,ab,kw**

**#4 HCC or hepatoma:ti,ab,kw**

**#5: #2 and #3 or #1 or #4**

**#6: microvascular or microvessel or microscopic or "portal vein":ti,ab,kw**

**#7: invasion or emboli or thrombi or thrombosis:ti,ab,kw**

**#8: microinvasion or microemboli or microthrombi or microthrombosis:ti,ab,kw**

**#9: #6 and #7 or #8**

**#10 margin or boundary or irregular or lobula* or confluen* or extension or infiltrat***

**#11 #5 and #6 and #10**

**Final record: 11 studies.**

**Table 1: Detailed characteristics of the 11 included studies**

| Study | Year | Country | P(non-smooth)( %) | Age | P (male)( %) | P(mvi+)(%) | Macro | Pre-Anti | Size (cm) | Surgery | Imaging |
| --- | --- | --- | --- | --- | --- | --- | --- | --- | --- | --- | --- |
| Kim *et al*.[^1^](#_ENREF_1) | 2009 | Korea | 39 | 55 | 88 | 50 | - | - | 3.6 | R | MR |
| Ariizumi *et al.*[*^2^*](#_ENREF_2) | 2011 | Japan | 39 | 67 | 80 | 18 | N | N | 2.9 | R | MR |
| Chou *et al.*[*^3^*](#_ENREF_3) | 2012 | China | 39 | 60 | 74 | 49 | N | N | 4.1 | R | CT |
| Witjes *et al*. [*^4^*](#_ENREF_4) | 2012 | Netherlands | 28 | 56 | 77 | 70 | N | Y | 5.8 | R/T | MR |
| Chou *et al.*[*^5^*](#_ENREF_5) | 2014 | China | 53 | 63 | 72 | 59 | N | N | 4.4 | R/T | CT |
| Xu *et al.* [*^6^*](#_ENREF_6) | 2014 | China | 18 | 53 | 87 | 36 | N | N | 1.4 | R | MR |
| Ahn *et al.*[*^7^*](#_ENREF_7) | 2015 | Korea | 40 | 52 | 82 | 25 | N | N | 3.1 | T | MR |
| Lei *et al.* [*^8^*](#_ENREF_8) | 2016 | China | 17 | 52 | 86 | 30 | N | N | 3.2 | R | MR |
| Renzulli *et al.*[*^9^*](#_ENREF_9) | 2016 | Italy | 59 | 62 | 81 | 64 | N | N | 3.4 | R | CT/MR |
| Wu *et al.*[*^10^*](#_ENREF_10) | 2016 | Japan | 38 | 70 | 80 | 19 | N | N | 4.0 | R | CT/MR |
| Yang *et al.*[*^11^*](#_ENREF_11) | 2016 | China | 39 | 56 | 86 | 32 | N | N | 3.0 | R | MR |

P(non-smooth)(%): percentage of patients with a non-smooth tumor margin on preoperative imaging test; P(male)( %): percentage of male patients; P(mvi+) (%): percentage of MVI positive patients; Macro: studies included patients with macrovascular invasion (Studies declaring curative treatment for included patients were considered as non-macrovascular); Pre-Anti: studies included patients with application of preoperative anti-tumor therapy; Imaging: imaging technology applied; -: unclear; Y: yes/positive; N: no/negative; R: resection; T: transplantation;

**Table 2: Results of Meta-regression**

| Possible source of bias | Coefficient | standard error | P | RDOR[95% CI] |
| --- | --- | --- | --- | --- |
| Age(≥60/<60) | 2.49 | 0.42 | <0.01 | 12.09[4.62, 31.61] |

# Table 3: Heterogeneity (I^2^) of pooled results

| Category | | Study number | SEN(%) | SPE(%) | PLR(%) | NLR(%) | | DOR(%) |
| --- | --- | --- | --- | --- | --- | --- | --- | --- |
| Summary | | 11 | 95.5 | 53.1 | 81.6 | 94.3 | | 89.1 |
| Age  (mean) | ≥60 | 5 | 46.9 | 46.2 | 33.2 | 44.2 | | 0 |
|  | <60 | 6 | 90.4 | 63.5 | 57.6 | 74.2 | 65.1 | |
| Imaging | CT | 2 | 71.7 | 0 | 0 | 71.1 | 37.2 | |
|  | MR | 7 | 91.6 | 59.8 | 74.6 | 78.3 | 71.8 | |

SEN: sensitivity; SPE: specificity; PLR: positive likelihood ratio; NLR: negative likelihood ratio; DOR: diagnostic odds ratio

# References:

1 Kim, H. *et al.* Can microvessel invasion of hepatocellular carcinoma be predicted by pre-operative MRI? *Eur Radiol* **19**, 1744-1751, doi:10.1007/s00330-009-1331-8 (2009).

2 Ariizumi, S. *et al.* A non-smooth tumor margin in the hepatobiliary phase of gadoxetic acid disodium (Gd-EOB-DTPA)-enhanced magnetic resonance imaging predicts microscopic portal vein invasion, intrahepatic metastasis, and early recurrence after hepatectomy in patients with hepatocellular carcinoma. *J Hepatobiliary Pancreat Sci* **18**, 575-585, doi:10.1007/s00534-010-0369-y (2011).

3 Chou, C. T. *et al.* Prediction of microvascular invasion of hepatocellular carcinoma by pre-operative CT imaging. *British Journal of Radiology* **85**, 778-783, doi:10.1259/bjr/65897774 (2012).

4 Witjes, C. D. *et al.* Histological differentiation grade and microvascular invasion of hepatocellular carcinoma predicted by dynamic contrast-enhanced MRI. *Journal of magnetic resonance imaging : JMRI* **36**, 641-647, doi:10.1002/jmri.23681 (2012).

5 Chou, C. T. *et al.* Prediction of microvascular invasion of hepatocellular carcinoma: preoperative CT and histopathologic correlation. *AJR Am J Roentgenol* **203**, W253-259, doi:10.2214/ajr.13.10595 (2014).

6 Qiu, D. M. *et al.* The expression of beclin-1, an autophagic gene, in hepatocellular carcinoma associated with clinical pathological and prognostic significance. *BMC cancer* **14**, 327, doi:10.1186/1471-2407-14-327 (2014).

7 Ahn, S. Y. *et al.* Prediction of microvascular invasion of hepatocellular carcinoma using gadoxetic acid-enhanced MR and (18)F-FDG PET/CT. *Abdom Imaging* **40**, 843-851, doi:10.1007/s00261-014-0256-0 (2015).

8 Lei, Z. *et al.* Nomogram for preoperative estimation of microvascular invasion risk in hepatitis B virus-related hepatocellular carcinoma within the milan criteria. *JAMA Surgery* **151**, 356-363, doi:10.1001/jamasurg.2015.4257 (2016).

9 Renzulli, M. *et al.* Can current preoperative imaging be used to detect microvascular invasion of hepatocellular Carcinoma?1. *Radiology* **279**, 432-442, doi:10.1148/radiol.2015150998 (2016).

10 Wu, T. H. *et al.* A non-smooth tumor margin on preoperative imaging predicts microvascular invasion of hepatocellular carcinoma. *Surgery Today*, 1-7, doi:10.1007/s00595-016-1320-x (2016).

11 Yang, C. *et al.* Microvascular invasion in hepatocellular carcinoma: is it predictable with a new, preoperative application of diffusion-weighted imaging? *Clinical imaging* **41**, 101-105, doi:10.1016/j.clinimag.2016.10.004 (2016).
